# Toll-Like Receptor 4 Decoy, TOY, Attenuates Gram-Negative Bacterial Sepsis

**DOI:** 10.1371/journal.pone.0007403

**Published:** 2009-10-09

**Authors:** Keehoon Jung, Jung-Eun Lee, Hak-Zoo Kim, Ho Min Kim, Beom Seok Park, Seong-Ik Hwang, Jie-Oh Lee, Sun Chang Kim, Gou Young Koh

**Affiliations:** 1 National Research Laboratory for Vascular Biology, Korea Advanced Institute of Science and Technology (KAIST), Daejeon, Republic of Korea; 2 Department of Biological Sciences, Korea Advanced Institute of Science and Technology (KAIST), Daejeon, Republic of Korea; 3 Department of Chemistry, Korea Advanced Institute of Science and Technology (KAIST), Daejeon, Republic of Korea; 4 Institute for the BioCentury, Korea Advanced Institute of Science and Technology (KAIST), Daejeon, Republic of Korea; 5 Graduate school of Nanoscience & Technology (WCU), Korea Advanced Institute of Science and Technology (KAIST), Daejeon, Republic of Korea; Institut de Pharmacologie et de Biologie Structurale, France

## Abstract

Lipopolysaccharide (LPS), the Gram-negative bacterial outer membrane glycolipid, induces sepsis through its interaction with myeloid differentiation protein-2 (MD-2) and Toll-like receptor 4 (TLR4). To block interaction between LPS/MD-2 complex and TLR4, we designed and generated soluble fusion proteins capable of binding MD-2, dubbed **T**LR4 dec**oy** receptor (TOY) using ‘the Hybrid leucine-rich repeats (LRR) technique’. TOY contains the MD-2 binding ectodomain of TLR4, the LRR motif of hagfish variable lymphocyte receptor (VLR), and the Fc domain of IgG1 to make it soluble, productive, and functional. TOY exhibited strong binding to MD-2, but not to the extracellular matrix (ECM), resulting in a favorable pharmacokinetic profile *in vivo*. TOY significantly extended the lifespan, when administered in either preventive or therapeutic manners, in both the LPS- and cecal ligation/puncture-induced sepsis models in mice. TOY markedly attenuated LPS-triggered NF-κB activation, secretion of proinflammatory cytokines, and thrombus formation in multiple organs. Taken together, the targeting strategy for sequestration of LPS/MD-2 complex using the decoy receptor TOY is effective in treating LPS- and bacteria-induced sepsis; furthermore, the strategy used in TOY development can be applied to the generation of other novel decoy receptor proteins.

## Introduction

Sepsis caused by Gram-negative bacterial infection is a life-threatening disease characterized by profound inflammatory responses, multi-organ dysfunction with marked thrombus formation, and a high mortality rate (∼60%) [1]. Lipopolysaccharide (LPS), the Gram-negative bacterial outer membrane glycolipid, induces sepsis through its interaction with LPS-binding protein (LBP) or CD14 prior to subsequent formation of a complex with myeloid differentiation protein-2 (MD-2) and Toll-like receptor 4 (TLR4) [2]–[12].

Human TLR4 contains a 608-residue extracellular domain, a single transmembrane domain, and a 187-residue intracellular domain [13]. Crystal structural analysis has shown that TLR4 adopts the characteristic horseshoe-like shape of the LRR superfamily, with N-terminal (amino acids 27–202), central (amino acids 203–348), and C-terminal (amino acids 349–582) domains [14], [15]. MD-2 binds to the concave surface of the N-terminal and central domains of TLR4 [14]. In addition to TLR4-bound MD-2, the MD-2 protein is also secreted into the extracellular milieu in a soluble form, which is present in circulating blood [16], [17]. MD-2 has a β-cup fold structure that forms a hydrophobic pocket for LPS binding [14], [18]. Binding of the LPS/MD-2 complex to TLR4 causes TLR4 dimerization, and results in the activation of NF-κB leading to acute and severe inflammation and sepsis [7]–[9], [19].

In this regard, blocking TLR4 signaling activation using a decoy receptor could be an effective way to prevent LPS- or Gram-negative bacteria-induced sepsis if applied prior to or after challenge. Although two research groups have tried to generate the extracellular domain of TLR4 protein as a decoy receptor for MD-2 [17], [20], it proved difficult to generate a substantial amount of the protein because it is insoluble, its production rate is extremely low, and it is hard to purify due to its intrinsic biochemical properties. Therefore, the trial of blocking TLR4 using a decoy receptor in preventing sepsis under *in vivo* conditions has not been achieved until now. To overcome these problems, we recently developed a novel method, ‘the Hybrid LRR Technique [14]’, to generate a massive amount of soluble extracellular domains of TLR4 protein. Variable lymphocyte receptors (VLRs) are a new type of immune receptors in jawless fish. These receptors resemble the adaptive immune receptors in jawed vertebrates. VLRs and TLRs commonly contain the LRR domain in the extracellular fragment, which is composed of a signal sequence, an N-terminal cap (LRRNT), several LRR modules, and a C-terminal cap (LRRCT) [14], [21]. Therefore, stable TLR4-VLR hybrid proteins can be generated in large amounts without any loss of the intrinsic structural integrity of TLR4 by replacing some LRR modules and LRRCT of TLR4 with those of VLR, termed TV3 and TV8 [14]. The TV3 and TV8 proteins are able to complex with MD-2 and Eritoran (a synthetic LPS antagonist) [22]. By fusion of the Fc domain of IgG1 to TV3 and TV8, we were able to generate dimeric TLR4 decoy receptor TOY, which is effective in treating LPS- and bacteria-induced sepsis.

## Results and Discussion

**Figure 1:**
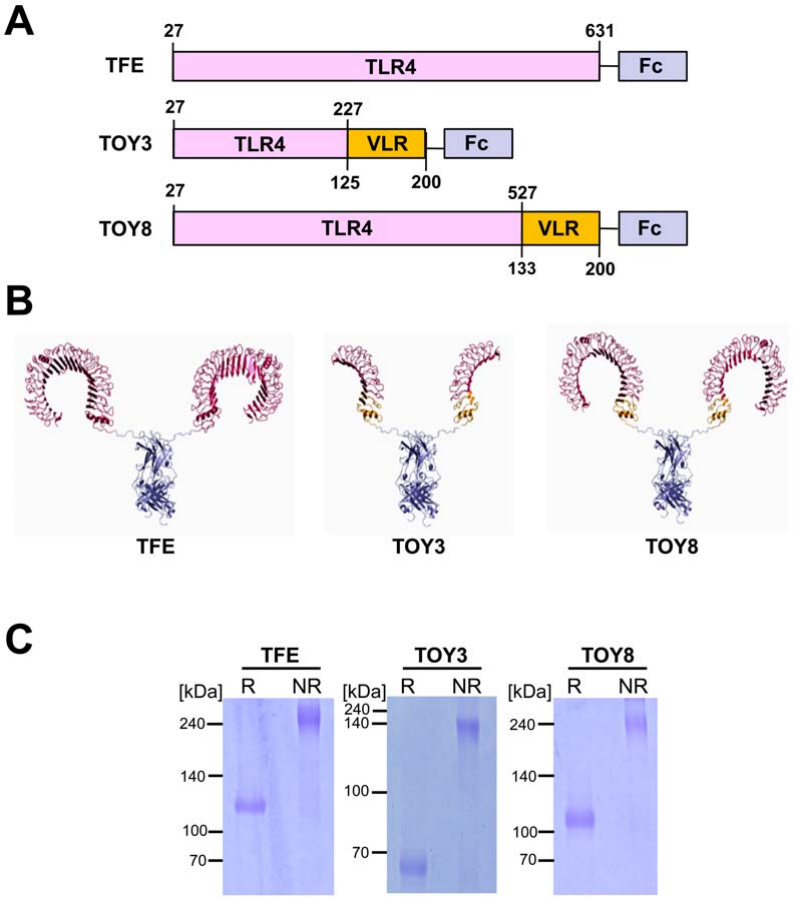
Structures of TFE and TOY constructs. (A) Schematic diagrams of the constructs showing the relative sizes of the human TLR4 ectodomain (TLR4), VLRB.61 fragment (VLR) of hagfish, and human IgG-Fc (Fc). Numbers indicate amino acids of the parental proteins. (B) Crystal structures based on computer modeling. The domains depicted are TLR4 (red), VLR (yellow), and Fc (blue). (C) Each 2 µg of reduced (R) and nonreduced (NR) proteins was separated by SDS-PAGE and stained with Coomassie blue. Molecular masses (kDa) are indicated at left.

**Figure 2:**
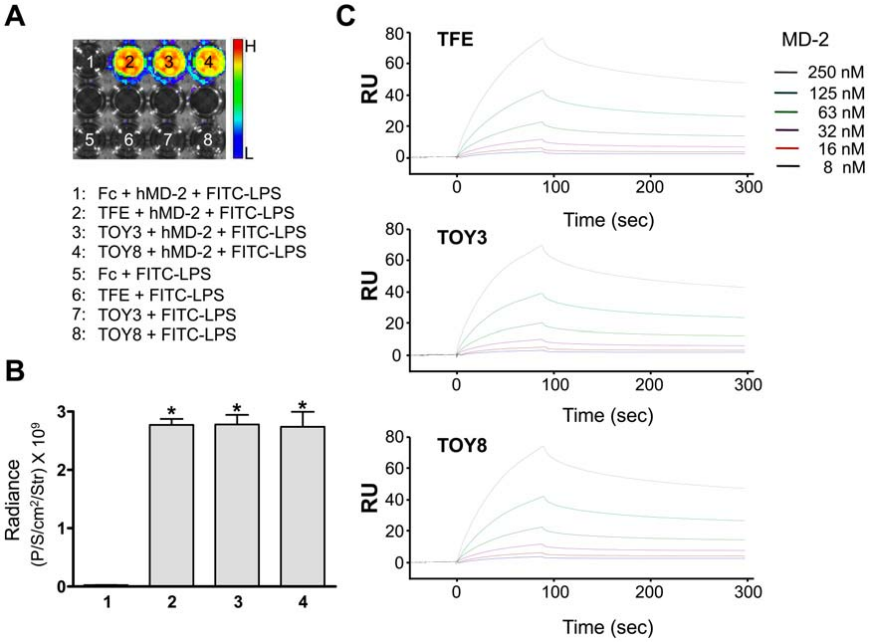
*In vitro* binding analysis reveals that LPS binds to TFE, TOY3, or TOY8 through MD-2 and BIAcore analysis of interaction between TFE, TOY3, or TOY8 and MD-2. (A) 200 ng of Fc, TFE, TOY3, or TOY8 were coated onto 96-well plates. FITC-labeled LPS (10 µg/ml) was incubated in the presence or absence of MD-2 (1 µg/ml) in each indicated well for 2 hr. The fluorescence signal was measured by IVIS imaging. (B) Fluorescence was quantified and expressed as radiance (photon/sec/cm^2^/steradian). Bars represent means ± S.D. (*n* = 4). *, *P*<0.05 *versus* Fc+hMD-2+FITC-LPS (1). The x-axis numbering represents the number of each well in (A). (C) Sensorgrams for the association and dissociation of MD-2 on immobilized TFE, TOY3, or TOY8. One microgram of TFE, TOY3, or TOY8 was immobilized on a Sensor Chip CM5 (BIAcore) using N-hydroxysuccinimide (NHS) and 1-ethyl-3(3-dimethylaminopropyl) carbodiimide (EDC) amine coupling reagent at approximately 2,000 resonance units (RU). As a control, BSA protein was immobilized on another portion of the same chip. Recombinant MD-2 proteins were then applied onto the immobilized TFE, TOY3, or TOY8 surfaces, and the amount captured was recorded in sensorgrams as RU. All samples were in running buffer to minimize bulk effects. The kinetic parameters of the binding interactions were calculated from the sensorgrams by nonlinear curve fitting using BIAEVALUATION software (BIAcore). RU represents resonance units.

**Figure 3:**
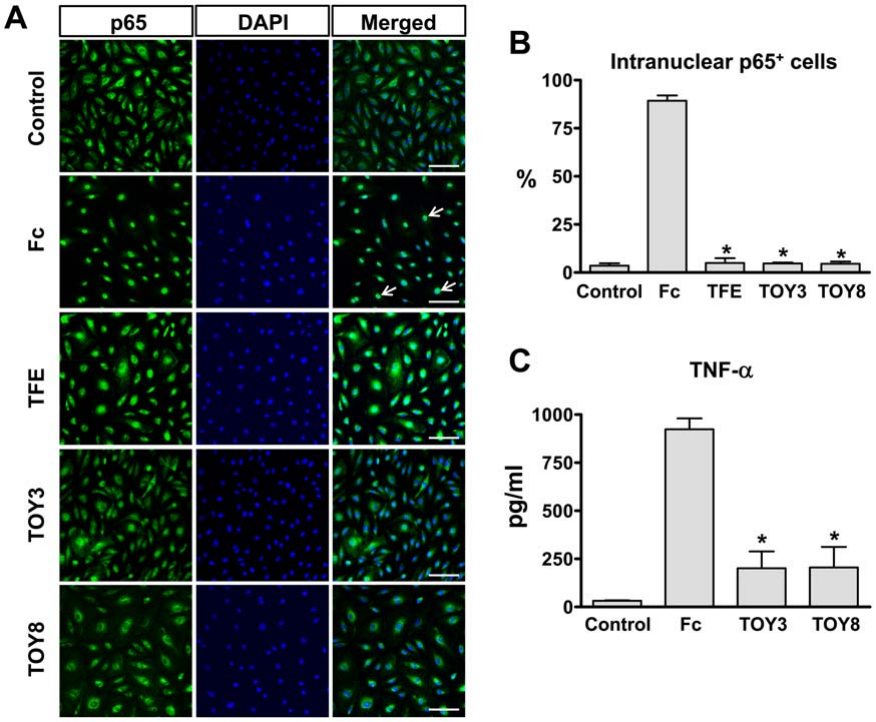
Pre-incubation of TFE, TOY3, and TOY8 markedly attenuates LPS-induced NF-κB activation in primary cultured lymphatic endothelial cells and TNF-α secretion in peritoneal macrophages. LEC (primary lymphatic microvascular endothelial cells derived from human adult dermis) were purchased from Cambrex Inc. (East Rutherford, NJ) and maintained in endothelial cell basal medium-2 with growth supplements (EBM-2 MV). Passage 4–6 LEC were incubated in EBM-2 MV containing 1 % FBS for 8 hr and then with 1 µg/ml of Fc, TFE, TOY3, or TOY8 for 15 min, and then the LEC were treated with LPS (500 ng/ml) for 30 min. (A) For determination of NF-κB activation, nuclear translocalization of p65 (a subunit of NF-κB) was analyzed by immunostaining (green). Nuclei were counterstained with DAPI (blue). Arrows indicate nuclear translocalization of p65. Scale bars, 100 µm. (B) Cells positive for p65 intranuclear staining (white arrows) were counted among 100 cells arbitrarily chosen in 4 different regions, and the values presented as a percentage of the total cell number. Bars represent means ± S.D. (*n* = 4). *, *P*<0.05 versus Fc. (C) Primary cultured macrophages from mouse peritoneal cavity were pre-treated with 1.0 µg/ml of Fc, TOY3, or TOY8 for 30 min, and then were treated with LPS (100 ng/ml) for 4 hr. Culture media were sampled, and levels of TNF-α were measured. Bars represent means ± S.D. (*n* = 5). *, *P*<0.05 versus Fc.

We fused human IgG-Fc to the carboxy-terminal portion of TV3 and TV8, and named the resulting products **T**LR4 dec**oy** receptor-3 and -8 (TOY3 and TOY8) (**Figure 1A**). As a control, we also produced a fusion of IgG-Fc to the **T**LR4 **f**ull-length **e**ctodomain (TFE; **Figure 1A**). We used computer modeling based on crystal structures to illustrate the potential structures of the three constructs (**Figure 1B**). We were able to produce the TFE, TOY3, and TOY8 proteins in CHO cells at rates of 0.1, ∼20, and ∼15 mg/L. The production rates of TOY3 and TOY8 are currently amplified by the methotrexate selection process [23]. The purified TFE, TOY3, and TOY8 under reducing conditions revealed predominantly single bands of the expected molecular masses of ∼110, ∼65 and, ∼105 kDa, respectively (**Figure 1C**). Under non-reducing conditions, the recombinant proteins were present as disulfide-linked dimers due to the presence of the Fc domain (**Figure 1C**). *In vitro* binding analysis revealed that TFE, TOY3, or TOY8 could interact not only with MD-2 but also with LPS/MD-2 complex (**Figure 2** and **Figure S1**). Surface plasmon resonance analyses revealed that all three proteins directly interacted with MD-2, and the *K*_D_ of TFE, TOY3, and TOY8 binding to MD-2 was ∼81, ∼76, and ∼56 nM (**Figure 2C**). Thus, the VLR component in TOY did not substantially alter the binding affinity for MD-2. *In vitro* analyses demonstrated that both TOY3 and TOY8 largely inhibited LPS-induced NF-κB activation in primary cultured lymphatic endothelial cells (**Figure 3A and 3B**) and also diminished LPS-induced TNF-α secretion in macrophages (**Figure 3C**).

**Figure 4:**
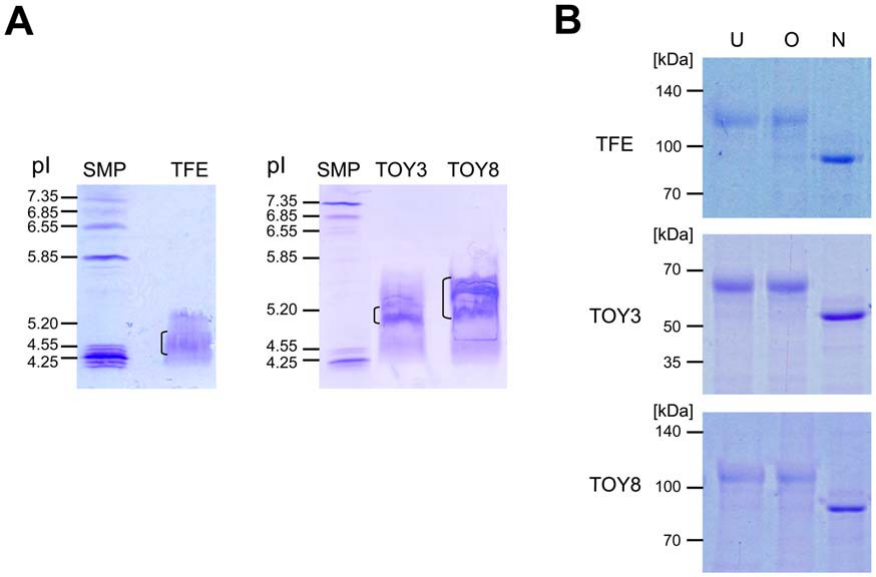
Isoelectric focusing and glycosylation analysis of TFE, TOY3, and TOY8. (A) 20 µg of the indicated protein was loaded on an IsoGel Agarose IEF Plate and run at 50 mA constant current for 3 hr. The gels were stained with Coomassie blue. Each bracket marks the pI range of the indicated protein. Reference pI values are indicated by the standard marker proteins (SMP). (B) 2 µg of the indicated protein was digested with O-glycosidase (O) and PNGase F (N) and was separated by SDS-PAGE and stained with Coomassie blue. Each undigested protein was used as control (U). Molecular masses (kDa) are indicated at left.

**Figure 5:**
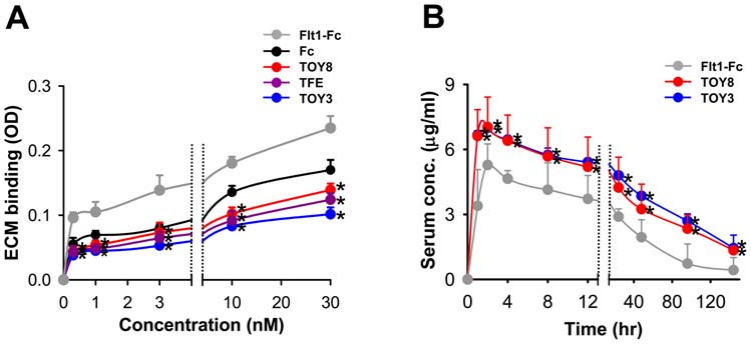
TOY3 and TOY8 have low binding affinity to ECM *in vitro* and display excellent pharmacokinetic profiles *in vivo*. (A) ELISA analysis depicting the binding affinity for different concentrations (0.3, 1, 3, 10, 30 nM) of each indicated protein to ECM. Values are given as means ± S.D. (*n* = 4). *, *P*<0.05 *versus* Fc. (B) Pharmacokinetic profiles. The indicated protein (5 mg/kg) was injected intraperitoneally into C57BL/6 mice. Then blood samples were taken from the tail vein at 1, 2, 4, 8, 12, 24, 48, 96, and 144 hrs, and serum levels of the proteins were measured by ELISA. Values are given as means ± S.D. (*n* = 4). *, *P*<0.05 *versus* Fc.

The isoelectric point (pI) of a recombinant protein affects its bioavailability and pharmacokinetics *in vivo*. High-pI proteins suffer from poor bioavailability because they adhere nonspecifically to the negatively charged proteoglycans of the extracellular matrix (ECM). While the theoretical pI values of TFE, TOY3, and TOY8 are 5.7, 11.8, and 6.0, their actual pI values are 5.0, 5.2, and 5.5, possibly due to abundant N-linked glycosylation (**Figure 4**). Indeed, the ECM binding assay demonstrated that TFE, TOY3, and TOY8 had minimal binding to ECM, whereas Flt1-Fc (pI 9.1) bound strongly to ECM (**Figure 5A**). To examine the *in vivo* pharmacokinetic profiles of the proteins, we performed a single intraperitoneal injection (5 mg/kg) of TOY3, TOY8, or Flt1-Fc recombinant protein into mice. TOY3 and TOY8 proteins were rapidly absorbed from the peritoneal cavity into systemic circulation. The proteins reached maximum levels in blood at 1∼2 hr after injection, and their half-lives (t_1/2_) were ∼2 days (**Figure 5B**). In mice (*n* = 4), TOY3 had a maximal concentration (*C*_max_) of 7.00±0.13 µg/ml and AUC (total “area under the curve of concentration”) of 20.50±0.96, TOY8 had a *C*_max_ of 7.03±0.85 µg/ml and AUC of 18.27±1.24, and Flt1-Fc had *C*_max_ of 5.28±0.49 µg/ml and AUC of 9.96±0.84. Thus, the pharmacokinetic profiles of the proteins were correlated to their *in vitro* ECM adhesion properties. TOY3 and TOY8 have relatively high bioavailability and excellent pharmacokinetic profiles *in vivo*, raising the possibility that TOY could block LPS/MD-2/TLR4 signaling *in vivo* by providing a decoy for TLR4.

**Figure 6:**
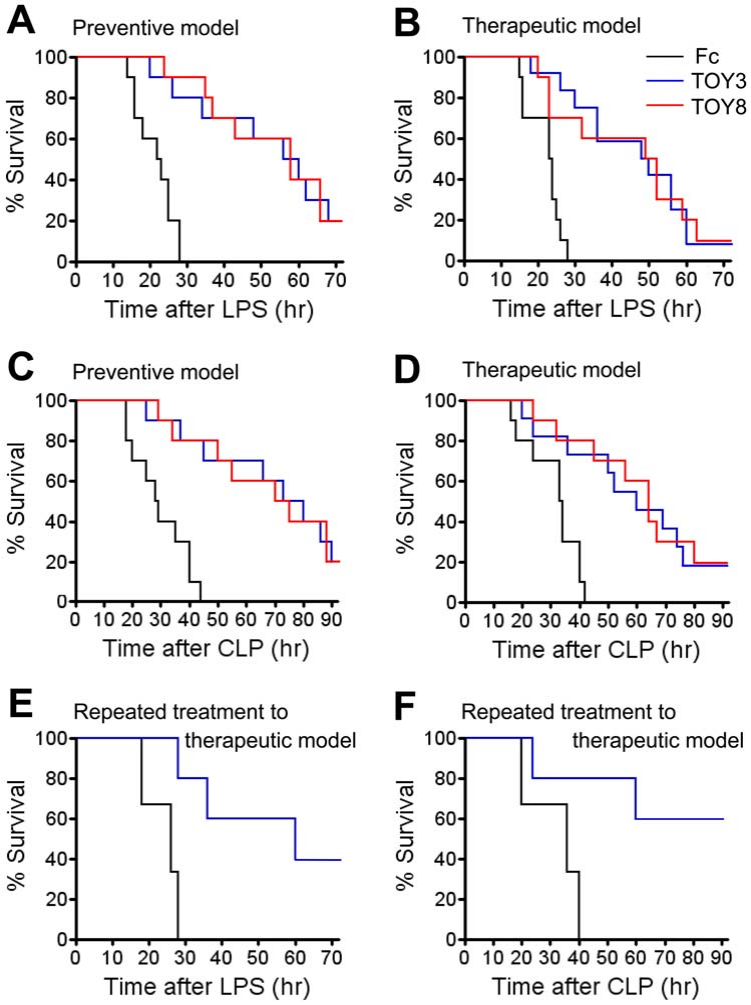
TOY attenuates lethality in two sepsis mouse models and is effective in both prophylactic and therapeutic treatments. (A–D) 20 mg/kg of Fc, TOY3, or TOY8 was given intraperitoneally to mice 30 min before (A) or 1 hr after (B) intraperitoneal administration of LPS (15 mg/kg). The protein is given 1 hr before (C) or 1 hr after (D) generation of CLP. (E and F) 20 mg/kg of Fc or TOY3 was given intraperitoneally to the mice at 1 hr and 12 hr after intraperitoneal administration of LPS (15 mg/kg) (E) or at 1 hr and 12 hr after generation of CLP (F). % Survival represents remaining live mice from total mice (*n* = 10–11).

**Figure 7:**
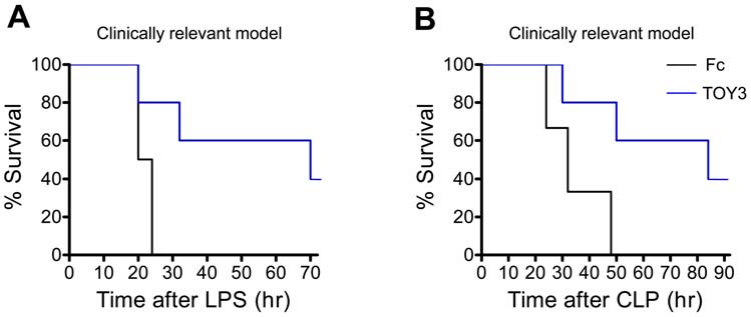
TOY ameliorates sepsis in clinically relevant models. (A and B) 20 mg/kg of Fc or TOY3 was given intraperitoneally to mice at 6 hr and 12 hr after intraperitoneal administration of LPS (15 mg/kg) (A) or generation of CLP (B). % Survival represents remaining live mice from total mice (*n* = 5).

To explore whether the TOY proteins could ameliorate sepsis *in vivo*, eight experiments were performed. For the first and second experiment, 20 mg/kg of TOY3 or TOY8 was given to mice at 30 min prior to or at 1 hr after administration of LPS (15 mg/kg) (‘preventive model’ or ‘therapeutic model’). Both TOY3- and TOY8-treated mice had an extended lifespan compared to Fc-treated mice (50% survival: ∼58 hr versus ∼22 hr, *P*<0.001 in the preventive model; ∼49 hr versus ∼22 hr, *P*<0.001 in the therapeutic model) (**Figure 6A and 6B**). For the third and fourth experiment, 20 mg/kg of TOY3 or TOY8 was given to the mice at 1 hr prior to or at 1 hr after generation of acute peritonitis by cecal ligation and puncture (CLP) (‘preventive model’ or ‘therapeutic model’). Both TOY3- and TOY8-treated mice had a prolonged lifespan compared to Fc-treated mice (50% survival: ∼74 hr versus ∼29 hr, *P*<0.001 in the preventive model; ∼60 hr versus ∼32 hr, *P*<0.001 in the therapeutic model) (**Figure 6C and 6D**). Thus, surprisingly, both TOY3 and TOY8 showed prominent preventive and therapeutic effects in the LPS- and CLP-induced sepsis models. For the fifth experiment, 20 mg/kg of TOY3 was given to the mice at 1 hr and 12 hr after administration of LPS (15 mg/kg) (‘repeated treatment to therapeutic model’). The TOY3-treated mice had a markedly extended lifespan compared to Fc-treated mice (50% survival: ∼60 hr versus ∼26 hr, *P*<0.001), and the 40% of TOY3-treated mice were completely rescued to live (**Figure 6E**). For the sixth experiment, 20 mg/kg of TOY3 was given to the mice at 1 hr and 12 hr after generation of CLP (‘repeated treatment to therapeutic model’). The repeated administration of TOY3 led to a dramatic increase in survival up to 96 hr after CLP (TOY3 versus Fc; 60% versus 0%, *P*<0.001) (**Figure 6F**). Thus, the repeated treatments with TOY3 markedly attenuated lethality in both the LPS- and CLP-induced sepsis models compared to the single treatment of TOY3 (50% survival: ∼60 hr versus ∼50 hr, *P*<0.01 in the LPS-induced sepsis model; 70% survival: ∼60 hr versus ∼50 hr, *P*<0.01 in the CLP-induced sepsis model). For the seventh and eighth experiments, 20 mg/kg of TOY3 was given to mice at 6 hr and 12 hr after administration of LPS (15 mg/kg) or the CLP procedure (‘clinically relevant model’). These repeated administrations of TOY3 also significantly improved survival compared to the controls in both the LPS- and CLP-induced sepsis models (50% survival: ∼69 hr versus ∼21 hr, *P*<0.001 in the LPS-induced sepsis model; ∼84 hr versus ∼32 hr, *P*<0.001 in the CLP-induced sepsis model) (**Figure 7A and 7B**). These repeated treatments with TOY3 markedly attenuated lethality in both the LPS- and CLP-induced sepsis models compared to the single treatment of TOY3 (50% survival: ∼70 hr versus ∼50 hr, *P*<0.01 in the LPS-induced sepsis model; ∼84 hr versus ∼60 hr, *P*<0.01 in the CLP-induced sepsis model). Therefore, repeated treatments with TOY3 could ameliorate sepsis even in a more clinically relevant situation.

**Figure 8:**
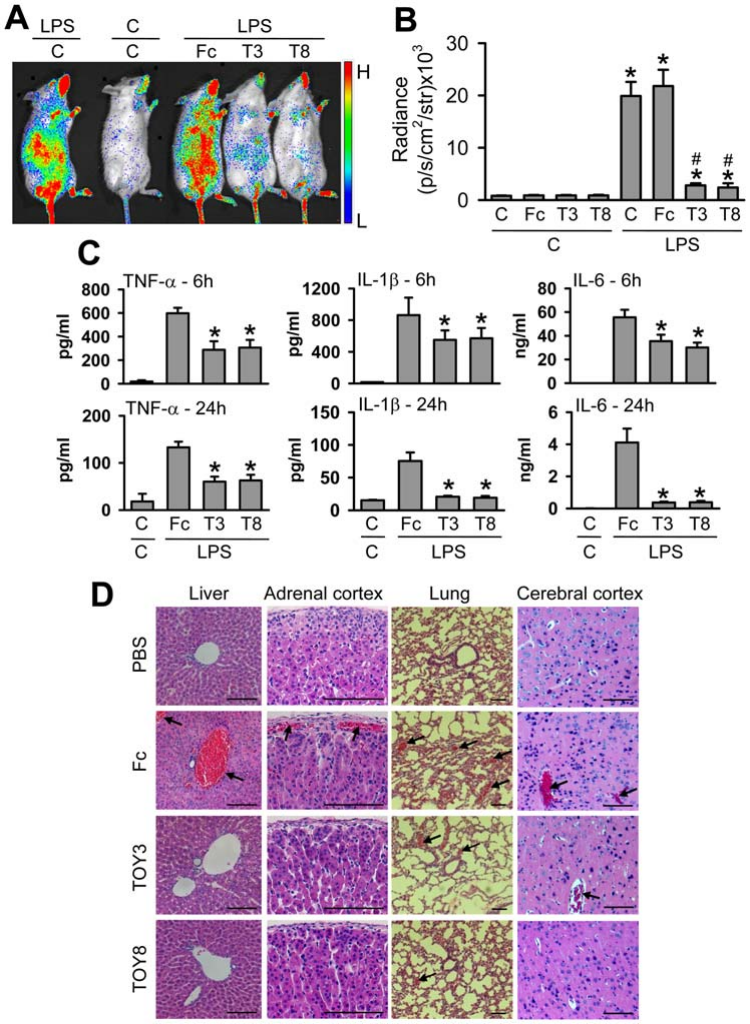
TOY attenuates LPS-induced NF-κB activation, secretion of proinflammatory cytokines, and thrombus formation. (A and B) *NF*κ*B-RE-luc* mice were injected intraperitoneally with control PBS (C) or 20 mg/kg of Fc, TOY3 (T3), or TOY8 (T8) at 1 hr after intraperitoneal administration of C or LPS (1 mg/kg). At 24 hr later, the luminescence signals from the whole body were examined by IVIS imaging (A). The luminescence was quantified and expressed as radiance (photon/sec/cm^2^/steradian) (B). Bars represent means ± S.D. (*n* = 3–4). *, *P*<0.05 *versus* C+C; ^#^, *P*<0.05 *versus* C+LPS or Fc+LPS. (C and D) Wild-type mice were injected intraperitoneally with C or 20 mg/kg of Fc, T3, or T8 at 1 hr after intraperitoneal administration of LPS (7.5 mg/kg) or C. At 6 and 24 hr later, plasma levels of TNF-α, IL-1β, and IL-6 were measured (C). Bars represent means ± S.D. (*n* = 4–5). *, *P*<0.05 *versus* Fc+LPS. At 24 hr after treatment, the mice were anesthetized, and samples of liver, adrenal gland, lung, and brain were H&E stained (D). Arrows indicate thrombi in the blood vessels. Scale bars, 100 µm.

Gram-negative bacterial sepsis is characterized by increased secretions of proinflammatory cytokines and thrombus formation in blood vessels, and both effects are brought through activation of NF-κB [2], [24]. To determine whether TOY attenuates NF-κB activation and subsequent secretion of proinflammatory cytokines and thrombus formation, we monitored these parameters in the LPS ‘therapeutic model’ using *NF*κ*B-RE-luc* [25] and wild-type mice. At 24 hr after LPS (1 mg/kg) administration, the luminescence signal examined by IVIS imaging system was markedly up-regulated in most regions of *NF*κ*B-RE-luc* mice, whereas the signal was barely detected in PBS-treated *NF*κ*B-RE-luc* mice (**Figure 8A and 8B**). Both TOY3 and TOY8 significantly attenuated the LPS-induced up-regulation of the signal in the *NF*κ*B-RE-luc* mice (**Figure 8A and 8B**). At 6 or 24 hr after LPS (7.5 mg/kg) administration, levels of TNF-α, IL-1β, and IL-6 in plasma and thrombi in the blood vessels of liver, adrenal cortex, lung, and brain were profoundly increased in the wild-type mice (**Figure 8C and 8D**). The mice treated with either TOY3 or TOY8 had markedly reduced levels of TNF-α, IL-1β, and IL-6 in plasma (**Figure 8C**), and fewer thrombi in the blood vessels (**Figure 8D**). Thus, TOY attenuated LPS-induced NF-κB activation, which increases the secretion of proinflammatory cytokines and results in thrombus formation in multiple organs.

In this study, we report for the first time the production of a TLR4 decoy receptor, TOY, which can be easily generated in large amounts. We also show for the first time that a modified decoy receptor of TLR4, TOY, is effective not only for prevention, but also for treatment of LPS- and bacteria-induced sepsis in mice. Because TOY would be used only once or twice during treatment of a life-threatening septic condition, the immune reaction against to TOY is not being considered. TOY3 and TOY8 produced almost identical effects in preventing sepsis; since TOY3 bears only the ‘A patch’ of the TLR4, this result implies that the ‘A patch’ of the TLR4 ectodomain is sufficient for MD-2 binding *in vivo* [14]. These findings give us additional structural information in TLR4 biology which shows that further application of TLR4 can be achieved with only the MD-2 minimal binding portion. Considering that TOY3 has a slightly higher production rate and smaller molecular size, it will be the most favorable construct for therapeutic use of TOY in the future. Recently, Roger et al. reported that a blocking antibody against the N-terminal and central domains of TLR4 also produced preventive and therapeutic effects in LPS- and bacteria-induced sepsis models [26]. Thus, targeting TLR4 either by the blocking antibody or decoy receptor such as TOY could be an amenable tool to relieve LPS- and bacteria-induced sepsis [26], [27]. In comparison, several lipid A analogs that are competitive inhibitors of LPS [22], [28] are able to target only LPS-free MD-2, but not LPS-bound MD-2, whereas TOY could sequester both free and bound forms of MD-2. Furthermore, since LBP and CD14 play accessory roles in LPS recognition by TLR4/MD-2 while MD-2 binds to LPS directly and induces TLR4 dimerization [8], [9], the efficacy of targeting TLR4/MD-2 using TOY could be different from that of blocking LBP [29] or CD14 [30]. Therefore, the effectiveness of TOY and blockers of LBP or CD14 should be carefully compared in future studies. Taken together, TOY would be an effective alternative therapeutic molecule for treatment of patients with bacterial sepsis, and our method provides a new platform biotechnology to generate novel decoy receptor from TLR proteins.

## Materials and Methods

### Generation of recombinant proteins

Gene constructs encoding different sizes of the ectodomain of human TLR4 [TFE (amino acid residues 27–631), TOY3 (amino acid residues 27–227), and TOY8 (amino acid residues 27–527)], the LRR module of hagfish VLR-B.61 (VLR), and the Fc domain of human IgG (Fc) were cloned into the pCMV-dhfr vector. Recombinant Chinese hamster ovary (rCHO) cells expressing TFE, TOY3, or TOY8 were established following a previously described method [23]. Briefly, the cells were established by transfection of a vector containing the dihydrofolate reductase (*dhfr*) and TFE, TOY3, or TOY8 gene into *dhfr*-deficient CHO cells (CRL-9096, American Type Culture Collection). This was followed by *dhfr*/methotrexate (MTX)-mediated gene amplification. Stable rCHO cells secreting the highest amount of TFE, TOY3, or TOY8 were selected with serially-increasing concentrations of MTX (0.001–0.08 µM, Sigma-Aldrich). Then the cells were grown and maintained in HyQ SFM4CHO (Hyclone) supplemented with 1% dialyzed fetal bovine serum (Invitrogen) and 0.08 µM MTX. After 4 days, the culture media containing recombinant proteins were harvested, and the recombinant TFE, TOY3, or TOY8 proteins were purified by using Protein-A sepharose affinity chromatography with subsequent acid elution and neutralization. After purification, each protein was quantitated using the Bradford assay and confirmed with Coomassie blue staining of an SDS-PAGE gel.

### *In vitro* binding assay

200 ng of BSA or MD-2 was coated onto 96-well plates. After blocking with 1% BSA for 1 hr, 40 µg/ml of Fc, TFE, TOY3, or TOY8 was incubated in each well for another 1 hr with or without FITC-labeled LPS (10 µg/ml). An HRP-conjugated anti-Fc antibody was incubated in each well for 1 hr, and then HRP substrate was added to each well. The fluorescence signal was measured by IVIS imaging, and the absorbance was measured by microplate reader (Bio-Rad).

### Surface plasmon resonance assay and isoelectric focusing

Binding between TFE, TOY3, or TOY8 and MD-2 were analyzed with the BIAcore 3000 (BIAcore AB). One microgram of TFE, TOY3, or TOY8 was immobilized on a Sensor Chip CM5 (BIAcore) using N-hydroxysuccinimide (NHS) and 1-ethyl-3(3-dimethylaminopropyl) carbodiimide (EDC) amine coupling reagent at approximately 2,000 resonance units (RU). As a control, BSA protein was immobilized on another portion of the same chip. Recombinant MD-2 proteins were then applied onto the immobilized TFE, TOY3, or TOY8 surfaces, and the amount captured was recorded in sensorgrams as RU. All samples were in running buffer to minimize bulk effects. The kinetic parameters of the binding interactions were calculated from the sensorgrams by nonlinear curve fitting using BIAEVALUATION software (BIAcore). To measure isoelectric points, 20 µg of each protein sample and standard marker proteins were loaded on an IsoGel Agarose IEF Plate pH 3–10 strip (Cambrex) and run at 50 mA constant current for 3 hr using 1 M phosphoric acid at the anode and 1 M sodium hydroxide at the cathode. The gels were stained with Coomassie blue.

### Glycosylation analysis of recombinant proteins

Two µg of TFE, TOY3, or TOY8 was incubated with O-glycosidase or PNGase F (QA-Bio) according to manufacturer's protocol, and then the protein was run on SDS-PAGE and the glycosylation pattern was analyzed by the migration shift after Coomassie blue staining.

### *In vitro* assays for NF-κB and TNF-α

NF-κB activity was assessed by immune-localization of p65 in nuclei of primary cultured lymphatic endothelial cells according to methods previously described [31]. Briefly, LEC (primary lymphatic microvascular endothelial cells derived from human adult dermis) were purchased from Cambrex Inc. (East Rutherford) and maintained in endothelial cell basal medium-2 with growth supplements (EBM-2 MV). Passage 4–6 LEC were incubated in EBM-2 MV containing 1 % FBS for 8 hr and then with 1 µg/ml of Fc, TFE, TOY3, or TOY8 for 15 min, and then the LEC were treated with LPS (500 ng/ml) for 30 min. Primary cultured macrophages from mouse peritoneal cavity were used to assay TNF-α secretion. Three days after intraperitoneal injection of 1 ml of 3% thioglycolate into 9-week-old male C57BL/6 mice, we harvested macrophages by peritoneal lavage with PBS. We incubated 1×10^6^ macrophages per experimental condition in RPMI 1640 (Lonza) supplemented with 10% dialyzed fetal bovine serum (Invitrogen). The macrophages were pre-incubated for 30 min with 1 µg/ml of Fc, TOY3, or TOY8, and then treated with LPS (100 ng/ml) for 4 hr. Culture media were sampled, and levels of TNF-α were measured by ELISA (R&D systems).

### ECM binding assay and pharmacokinetic analysis

ECM-coated 96-well plates (Becton Dickinson) were used for ECM binding assays. Each recombinant protein (100 µg of Fc, TOY3, or TOY8) was injected subcutaneously into 8-week-old male C57BL/6 mice (∼25 g body weight), then the amount of each recombinant protein in blood was measured at the indicated times by sandwich ELISA.

### Animals

Specific pathogen-free 8–9-week-old male C3H/HeN mice and *NF*κ*B-RE-luc* [25] (Balb/C) mice were used. Animal care and experimental procedures were performed under approval from the Animal Care Committees of KAIST. To generate the LPS-induced sepsis model, mice were injected intraperitoneally with 15 mg/kg of LPS (*E. coli* O111:B4; List Biological Laboratories). To generate the CLP-induced sepsis model, mice were anesthetized, and ∼75% of the cecum was ligated and punctured with a 21-gauge needle. The mice received 20 mg/kg of Fc, TOY3, or TOY8 into the peritoneal cavity prior to or after the generation of sepsis.

### Monitoring NF-κB activation, measurement of proinflammatory cytokines, and histology

*NF*κ*B-RE-luc* [25] and wild-type mice were given 1.0 and 7.5 mg/kg of LPS 1 hr prior to administration of 20 mg/kg of Fc, TOY3, or TOY8. At 24 hrs after the LPS administration, the luminescence signal was examined by the IVIS imaging system (Xenogen). Plasma was sampled at 6 and 24 hrs, and levels of TNF-α, IL-1β, and IL-6 were measured by ELISA (R&D systems). Mice were sacrificed 24 hours after LPS treatment, and their livers, adrenal glands, lungs, and brains were harvested for histological studies. Harvested organs were fixed using 4% PFA dissolved in PBS at 4°C overnight and embedded in paraffin blocks. Sections (4-µm) were stained with H&E and analyzed under a phase-contrast light microscope.

### Statistics

Values are presented as means ± S.D. Significant differences between means were determined by analysis of variance followed by the Student-Newman-Keuls test. For analysis of survival curves, a log-rank test was performed.

## Supporting Information

Figure S1 In vitro binding analysis reveals that TFE, TOY3, or TOY8 could interact not only with MD-2 but also with LPS/MD-2 complex. (A) BSA or MD-2 was coated onto 96-well plates and 40 µg/ml of Fc, TFE, TOY3, or TOY8 was incubated in each well with or without FITC-labeled LPS. An HRP-conjugated anti-Fc antibody was incubated in each well, and then HRP substrate was added. The fluorescence signal of each well is shown. (B) Fluorescence and absorbance were measured. Fluorescence is expressed as radiance (photon/sec/cm2/steradian) on the left y-axis, and absorbance is shown on the right y-axis. Bars represent means ± S.D. (n = 4). *, P<0.05 versus hMD-2+Fc (5); #, P<0.05 versus hMD-2+FITC-LPS+Fc (9). The x-axis numbering represents the number of each well in (A).
(1.28 MB TIF)
