# Supplementary figures and images for: Toll-Like Receptor 4 Decoy, TOY, Attenuates Gram-Negative Bacterial Sepsis
Source: PLoS One. 2009 Oct 9;4(10):e7403. doi: 10.1371/journal.pone.0007403 (PMC2754608; doi:10.1371/journal.pone.0007403)

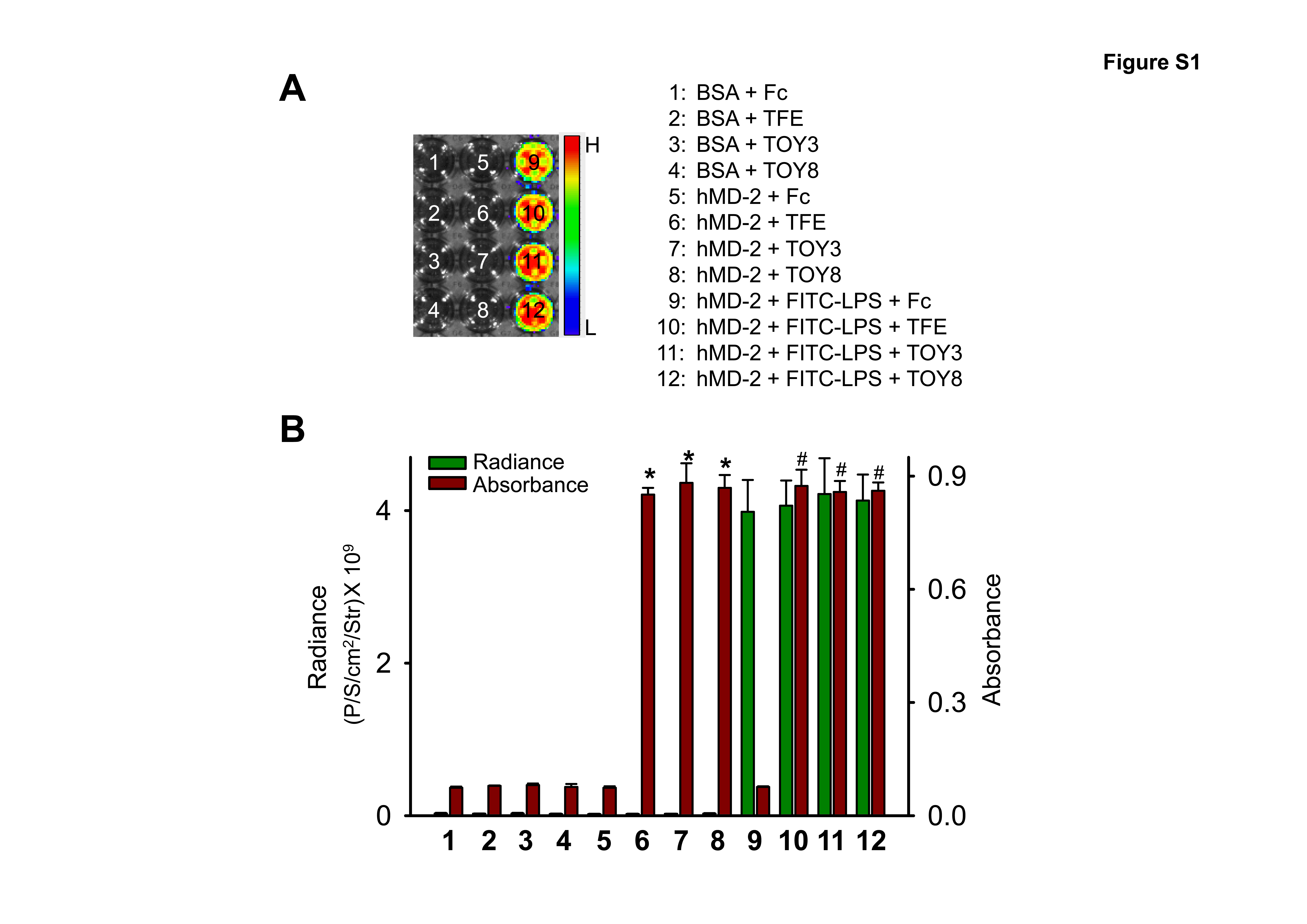

Supplement: Figure S1 — In vitro binding analysis reveals that TFE, TOY3, or TOY8 could interact not only with MD-2 but also with LPS/MD-2 complex. (A) BSA or MD-2 was coated onto 96-well plates and 40 µg/ml of Fc, TFE, TOY3, or TOY8 was incubated in each well with or without FITC-labeled LPS. An HRP-conjugated anti-Fc antibody was incubated in each well, and then HRP substrate was added. The fluorescence signal of each well is shown. (B) Fluorescence and absorbance were measured. Fluorescence is expressed as radiance (photon/sec/cm2/steradian) on the left y-axis, and absorbance is shown on the right y-axis. Bars represent means ± S.D. (n = 4). *, P<0.05 versus hMD-2+Fc (5); #, P<0.05 versus hMD-2+FITC-LPS+Fc (9). The x-axis numbering represents the number of each well in (A). (1.28 MB TIF) [file pone.0007403.s001.tif]
